# A Novel Four-Gene Signature Associated With Immune Checkpoint for Predicting Prognosis in Lower-Grade Glioma

**DOI:** 10.3389/fonc.2020.605737

**Published:** 2020-10-30

**Authors:** Youchao Xiao, Gang Cui, Xingguang Ren, Jiaqi Hao, Yu Zhang, Xin Yang, Zhuangzhuang Wang, Xiaolin Zhu, Huan Wang, Chunyan Hao, Hubin Duan

**Affiliations:** ^1^Department of Neurosurgery, First Hospital of Shanxi Medical University, Taiyuan, China; ^2^Department of Neurosurgery, The Third Affiliated Hospital of Shandong First Medical University (Affiliated Hospital of Shandong Academy of Medical Sciences), Jinan, China; ^3^Department of Neurosurgery, General Hospital of TISCO, Taiyuan, China; ^4^Department of Geriatrics, First Hospital of Shanxi Medical University, Taiyuan, China; ^5^Department of Neurosurgery, Lvliang People’s Hospital, Lvliang, China

**Keywords:** gene signature, prognostic value, biomarker, immune checkpoint, cancer stem cell, isocitrate dehydrogenase mutation

## Abstract

The overall survival of patients with lower grade glioma (LGG) varies greatly, but the current histopathological classification has limitations in predicting patients’ prognosis. Therefore, this study aims to find potential therapeutic target genes and establish a gene signature for predicting the prognosis of LGG. CD44 is a marker of tumor stem cells and has prognostic value in various tumors, but its role in LGG is unclear. By analyzing three glioma datasets from Gene Expression Omnibus (GEO) database, CD44 was upregulated in LGG. We screened 10 CD44-related genes *via* protein–protein interaction (PPI) network; function enrichment analysis demonstrated that these genes were associated with biological processes and signaling pathways of the tumor; survival analysis showed that four genes (CD44, HYAL2, SPP1, MMP2) were associated with the overall survival (OS) and disease-free survival (DFS)of LGG; a novel four-gene signature was constructed. The prediction model showed good predictive value over 2-, 5-, 8-, and 10-year survival probability in both the development and validation sets. The risk score effectively divided patients into high- and low- risk groups with a distinct outcome. Multivariate analysis confirmed that the risk score and status of IDH were independent prognostic predictors of LGG. Among three LGG subgroups based on the presence of molecular parameters, IDH-mutant gliomas have a favorable OS, especially if combined with 1p/19q codeletion, which further confirmed the distinct biological pattern between three LGG subgroups, and the gene signature is able to divide LGG patients with the same IDH status into high- and low- risk groups. The high-risk group possessed a higher expression of immune checkpoints and was related to the activation of immunosuppressive pathways. Finally, this study provided a convenient tool for predicting patient survival. In summary, the four prognostic genes may be therapeutic targets and prognostic predictors for LGG; this four-gene signature has good prognostic prediction ability and can effectively distinguish high- and low-risk patients. High-risk patients are associated with higher immune checkpoint expression and activation of the immunosuppressive pathway, providing help for screening immunotherapy-sensitive patients.

## Introduction

The central nervous system’s primary tumors are dominated by gliomas with histologic characteristics of normal glial cells and are often named after these similarities. According to the classification criteria for the central nervous system's tumors, gliomas were divided into four grades according to the pathological characteristics of gliomas, in which grade I/II is the low grade and grade III/IV is the high grade (1, 2). Because grade II and grade III gliomas are similar in many ways and are less malignant than the glioblastoma (grade IV), grade II/III gliomas are called lower grade gliomas (LGG) (3–5).

For decades, the criteria for diagnosing and classifying brain tumors have been microscopic or histopathological features, and the WHO grade system is commonly used for prognostic prediction in glioma patients (1, 2). The histological features are subject to inter-observer variability, leading to an ambiguous diagnosis and inaccurate prognostic prediction in gliomas (6–9). The prognostic prediction in glioma patients may be complicated, and the prognosis in patients with the same WHO grade glioma can vary dramatically (10). Therefore, gene expression profiles and molecular markers have been applied in clinical practice for objective diagnosis, specific classification, and accurate clinical outcomes (11–14). To the best of our knowledge, surveys for the classification and prognosis prediction of gliomas are mainly focused on high-grade gliomas or glioblastoma; biomarkers associated with stratification of prognosis in patients with LGG are still limited.

Emerging evidence showed that cancer stem cells (CSCs) play an essential role in tumor progression, metastasis, recurrence, and poor clinical outcome (15–17). CD44 gene is a common marker of CSC and shown to express in many tumors to play a significant role in cell growth, survival, tumor proliferation, metastasis, resistance (18, 19). CD44 also participates in multiple signaling pathways, including the Hippo-Yap signaling pathway (20), Wnt/*β*-catenin pathway (21, 22), and lymphocyte activation pathway (23). The expression of the CD44 gene was associated with poor prognosis in many cancers, such as non-small cell lung cancer (24), ovarian cancer (25), renal cell carcinoma (26), breast cancer (27). However, there is no consensus on the relationship between CD44 expression and prognosis in glioma patients (28–33).

Therefore, we attempt to explore CD44 gene expression in LGG and screen the prognostic genes of LGG. Then, we try to construct a CD44-related gene signature for LGG by screening genes associated with prognosis, validate the gene signature in the external validation set. Finally, we attempt to elucidate the association between CD44-related gene signature and immune function to develop tools for predicting the prognosis of LGG.

## Materials and Methods

### Identification of CD44 as Differentially Expressed Gene

**Figure 1:**
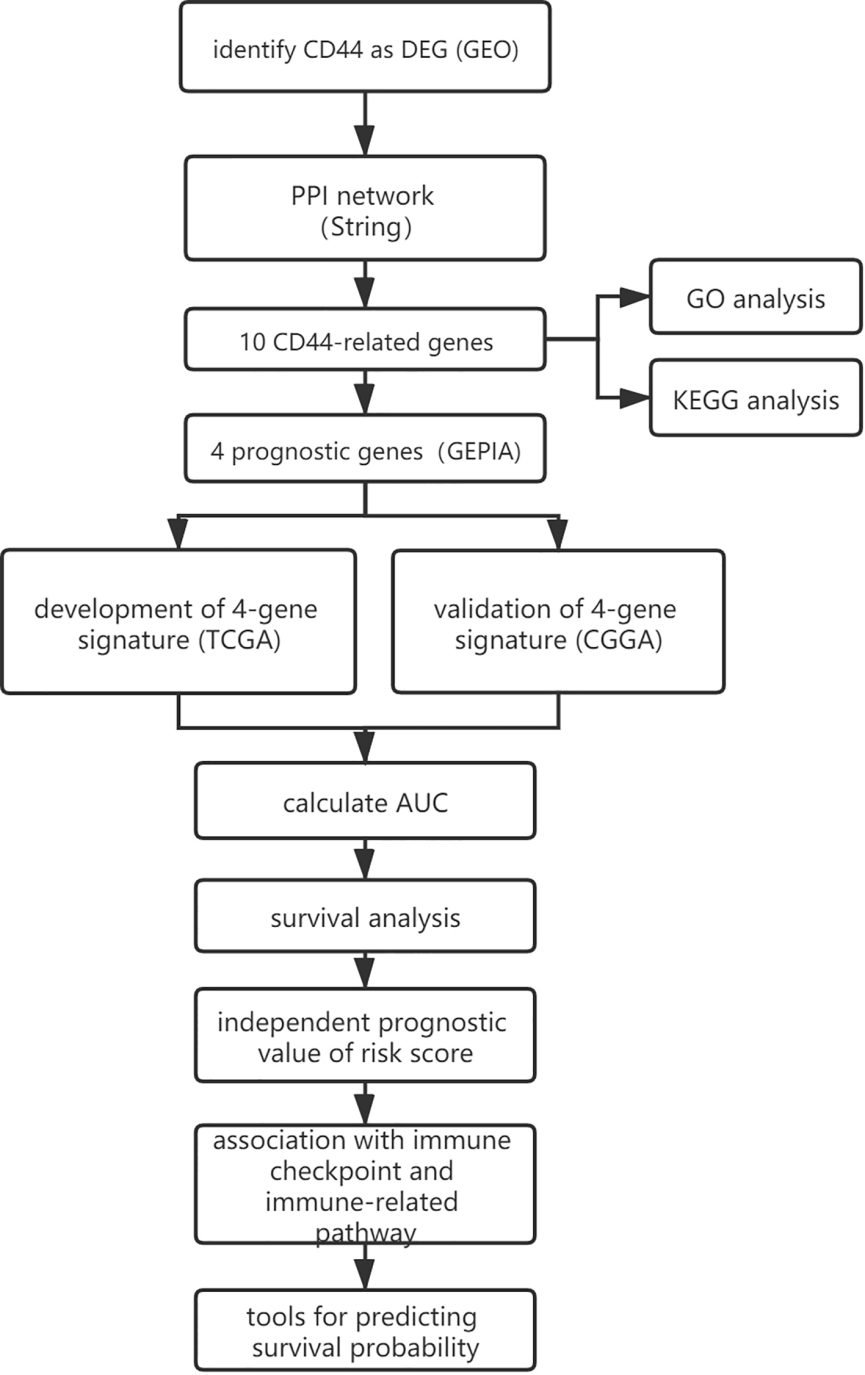
The workflow of the present study.

A flowchart of this study was presented in **Figure 1**. GEO (https://www.ncbi.nlm.nih.gov/geo/) is a non-profit public database, and the gene expression profiles of three datasets (34–36) were downloaded from GEO, including GSE4290, GSE109857, GSE15824. These three datasets contained 33 normal brain samples and 219 LGG samples, and datasets were annotated according to the corresponding platform; **Table 1** has shown the detail information of datasets. To determinate whether CD44 is a DEG between LGG and normal brain samples, we used the limma package (37, 38) R software (R version 4.0.2) to analyze data extracted from datasets; the gene with |log2 fold change (FC)|>2 and adjusted p-value <0.05 was regarded as DEG.

**Table 1:** Details of three datasets.

Dataset	Normal brain sample	Lower-grade glioma		Platform
		II	III	
GSE4290	23	42	31	GPL570 [HG-U133_Plus_2] Affymetrix Human Genome U133 Plus 2.0 Array
GSE109857	5	97	34	GPL6480 Agilent-014850 Whole Human Genome Microarray 4x44K G4112F
GSE15824	5	7	8	GPL570 [HG-U133_Plus_2] Affymetrix Human Genome U133 Plus 2.0 Array
Total	33	146	73	

### PPI Network and Enrichment Analysis

To screen CD44-related genes, we used STRING (version 11.0; http://string.embl.de/) (39), a biological database and web resource that predicts comprehensive interactions of genes at the protein level, to explore CD44-related genes. The protein–protein interactions with medium confidence >0.9 were regarded as significant, and corresponding genes were identified as CD44-related genes.

The Database for Annotation, Visualization and Integrated Discovery (DAVID, version 6.8, https://david.ncifcrf.gov/) database (40, 41) was used to conduct the enrichment analysis of the CD44 gene and CD44-related genes. Enrichment analysis includes gene ontology (GO) analysis and Kyoto Encyclopedia of Genes and Genomes (KEGGs) pathway analysis, and GO analysis classifies gene functions into three categories, including cellular components (CCs), biological processes (BPs), and molecular functions (MFs). P < 0.05 was set as the cut-off criterion.

### Screen the Prognostic Genes by Using Gene Expression Profiling Interactive Analysis

GEPIA is an interactive web application based on The Cancer Genome Atlas (TCGA) and Genotype-Tissue Expression data (42). We used the GEPIA to screen prognostic genes from CD44 and CD44-related genes. Genes would be identified as prognostic genes when the gene met specific criteria, and the criteria were listed as the following: (a) gene expression level was significantly different in normal brain samples and lower grade gliomas; (b) gene was significantly associated with overall survival of LGG; (c) gene was significantly associated with disease-free survival of LGG. P-value <0.05 was regarded as significant.

### Development and Validation of the Gene Signature

The entire LGG cohort from the TCGA database (525 samples) was used as the development set and internal validation set; the external validation set contained 420 LGG samples from the Chinese Glioma Genome Atlas (CGGA) database, and the clinical characteristics of two cohorts were shown in **Table 2**. Identified prognostic genes were submitted to the multivariate Cox regression model to calculate each prognostic gene’s coefficient. For every patient, the risk score was calculated by the following equation:

$$\text{risk score }=\;\beta_{1}*{\text{gene}}_{1}+\beta_{2}*{\text{gene}}_{2}+\beta_{n}*{\text{gene}}_{n}$$

**Table 2:** Clinical characteristics of lower-grade glioma cohorts.

Clinical characteristic		TCGA (n = 525)	CGGA (n = 420)
Age (median, range)		(40, 14–87)	(40, 11–72)
	<60	455	403
	≥60	70	16
	Unknown	0	1
Gender	Female	238	185
	Male	287	235
Race	White	484	–
	Other	31	–
	Unknown	10	–
Grade	II	258	172
	III	266	248
	Unknown	1	0
Primary/recurrent	Primary	–	271
	Recurrent	–	149
Radiation therapy	No	174	99
	Yes	284	308
	Unknown	67	13
Chemical therapy	No	–	129
	Yes	–	281
	Unknown	–	10
IDH mutation	No	34	94
	Yes	91	288
	Unknown	400	38
MGMT methylation	No	–	129
	Yes	–	200
	Unknown	–	91
Motor change	No	355	–
	Yes	122	–
	Unknown	48	–
Sensor change	No	392	–
	Yes	72	–
	Unknown	61	–
Seizure history	No	183	–
	Yes	309	–
	Unknown	33	–
Headache history	No	301	–
	Yes	175	–
	unknown	49	–

IDH, isocitrate dehydrogenase; MGMT, O6-methylguanine-DNA methyltransferase; －, not reported.

Gene_n_ represents the expression value of the gene, and *β*_n_ represents the coefficient of the corresponding gene. According to the train set’s median risk score, LGG patients were classified into either a high-risk group or low-risk group. The area under the receiver operating characteristic curve (AUC) was used to estimate the risk model’s sensitivity and specificity. Kaplan–Meier curves were plotted, and log-rank tests were conducted to evaluate the gene signature’s prognostic value in the train set and validation set, respectively.

The performance of the risk model was validated by internal validation and external validation; internal validation was performed by bootstrap Cox proportional regression analysis based on 1,000 bootstrap samples, and external validation was conducted based on LGG patients from the CGGA database. Graphpad was used to conduct and visualize the risk score analysis of train set and validation set; risk score analysis included risk score distribution, survival status, and gene expression heatmaps.

### Independent Prognostic Value of Risk Model

Univariate analysis and multivariate analysis were performed to estimate the risk model’s independent prognostic value in the LGG cohort. In the train set, covariables included risk score, age, gender, race, grade, radiotherapy, isocitrate dehydrogenase (IDH) mutant, motor function change, sensor function change, seizer, headache. In the external validation set, covariables included risk score, age, gender, grade, radiotherapy, chemotherapy, IDH mutant, O6-methylguanine-DNA methyltransferase (MGMT) methylation. HR >1 indicates a favorable prognosis; HR <1 indicates an unfavorable prognosis. Factors with a p < 0.05 were identified as independent prognostic factors.

### Relationship Between the Gene Signature and LGG Subgroups

From the view of molecular parameters, LGG (Grade II/III gliomas) was divided into three subgroups based on IDH status and 1p/19q codeletion status, including oligodendroglioma (IDH mutant plus 1p/19q codeletion), astrocytoma (IDH mutant), and astrocytoma (IDH wild type) (2). The TCGA database divided LGG patients into three groups, including astrocytoma, oligodendroglioma, and mixed glioma (also known as oligoastrocytoma) (43, 44). In fact, most oligoastrocytomas can be re-diagnosed as oligodendroglioma or astrocytoma by genetic testing, and only a few are genuinely oligodendrogliomas (45, 46). The diagnosis of oligoastrocytoma is highly inadvisable (2). TCGA database did not provide the information of 1p/19q codeletion, and the CGGA database provided information on IDH status and 1p/19q codeletion status. Therefore, we were able to divide the LGG cohort from the CGGA database into three groups, which were listed as follows: LGG with IDH wild type, LGG with IDH mutant and 1p/19q non-codeletion (intact), and LGG with IDH mutant and 1p/19q codeletion. Therefore, we analyzed the LGG cohort from the CGGA database to explore the relationship between gene signature and three LGG subgroups. The expression of four genes and the value of risk score within three LGG subgroups were compared using analysis of variance (ANOVA) test. Kaplan–Meier curves were plotted, and log-rank tests were conducted to evaluate the gene signature’s prognostic value in these three LGG subgroups. P-value <0.05 was regarded as statistically significant, and p-value <0.1 indicates a statistically significant trend toward.

### Correlation Between Risk Score and Immune Checkpoint, Immune-Related Pathway

Immune checkpoint mainly includes Programmed cell death protein 1 (PD1), Programmed cell death 1 ligand 1 (PD-L1), Cytotoxic T Lymphocyte Antigen 4 (CTLA4), Lymphocyte-activation gene 3 (LAG3). Graphpad was used to visualize the gene expression profile of immune checkpoints in LGG cohorts; then, we explore the relationship between risk score and immune checkpoint by testing the difference of gene expression level in high- and low-risk groups. The results were shown as median–95% confidence interval upper bound. Differences between the two groups were assessed using the Mann–Whitney test. P < 0.05 was considered statistically significant.

Gene Set Enrichment Analysis (GSEA) (47) is a computational method identifying differentially activated signaling pathways in phenotypes of LGG. First, high- and low-risk phenotypes were defined according to the median of risk scores; then, GSEA produced an ordered list of genes based on the correlation between all genes and risk score; lastly, GSEA elucidated the significant survival difference observed between two phenotypes; gene set was permutated 1,000 time in every analysis. Nominal p-value < 0.05, false discovery rate (FDR) < 0.05, and normalized enrichment scores were taken to determine differentially activated signaling pathways in phenotypes.

### Tools for Predicting Survival Probability of LGG Patients

We attempted to develop tools for predicting survival probability or death probability at a special time, including 2-, 5-, 8-, and 10-year. The following equation calculated survival probabilities at certain years:

$$\text{S (t)}={\text{S0(t) }}^{\wedge }\text{ exp }(\text{risk score}).$$

S(t) means the survival probability at a specific time, S0(t) means the basic survival probability, t means the time. Therefore, we attempt to develop an excel table and a nomogram. The nomogram was developed by using the “rms” package.

## Result

### The Result of DEG Screening

**Figure 2:**
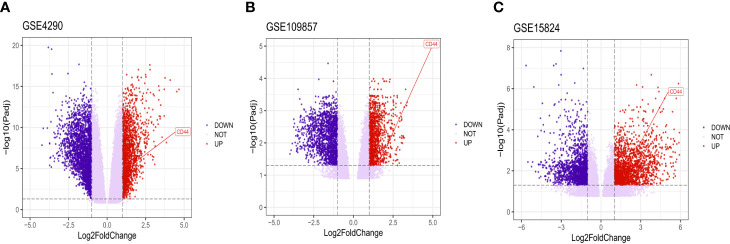
Volcano plot of all DEGs. CD44 gene was marked. The X-axis represents log2 fold change, and the Y-axis represents the log-transformed adjusted P values.

After analyzing the data to screen DEG *via* limma package R software, then we used the Volcano plot (**Figure 2**) to visualize the result of DEG screening *via* ggplot2 package (48) R software; we observed that the CD44 gene was an upregulated DEG with log2 FC>2 and adjusted p-value <0.05, log2 FC >2 suggested that CD44 played a role in oncogenicity.

### PPI Network and Enrichment Analysis of Genes

**Figure 3:**
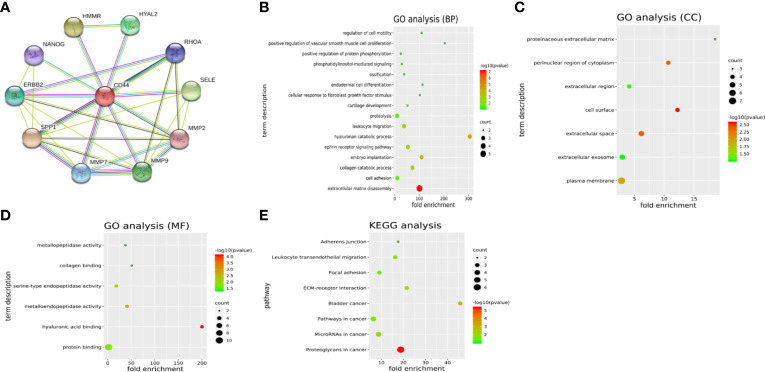
**(A)** PPI network of CD44 and CD44-related genes. **(B–D)** GO analysis of CD44 and CD44-related genes. **(E)** KEGG pathway analysis of CD44 and CD44-related genes. PPI, protein–protein interaction; BPs, biological processes; CCs, cellular components; MFs, molecular functions; GO, gene ontology; KEGG, Kyoto Encyclopedia of Genes and Genomes.

Another 10 genes were identified as CD44-related genes with significant interaction, including MMP7, MMP9, MMP2, SELE, RHOA, HYAL2, HMMR, NANOG, ERBB2, and SPP1. The PPI network of CD44 and CD44-related genes was constructed and visualized by the online STRING database (**Figure 3A**).

GO analysis showed that CD44 and its related genes were significantly enriched in the category of BP (**Figure 3B**), including extracellular matrix disassembly, cell adhesion, collagen catabolic process, embryo implantation. In the category of CC (**Figure 3C**), these genes were enriched in plasma membrane, extracellular exosome, extracellular space, and cell surface. In the category of MF (**Figure 3D**), genes were enriched in protein binding, hyaluronic acid binding, metalloendopeptidase activity, and serine-type endopeptidase activity. What is more, KEGG pathway analysis showed that these genes were mainly enriched in eight pathways (**Figure 3E**), including proteoglycans in cancer, microRNAs in cancer, pathways in cancer, bladder cancer, ECM-receptor interaction, focal adhesion, leukocyte transendothelial migration, and adherens junction.

### Prognostic Genes

**Figure 4:**
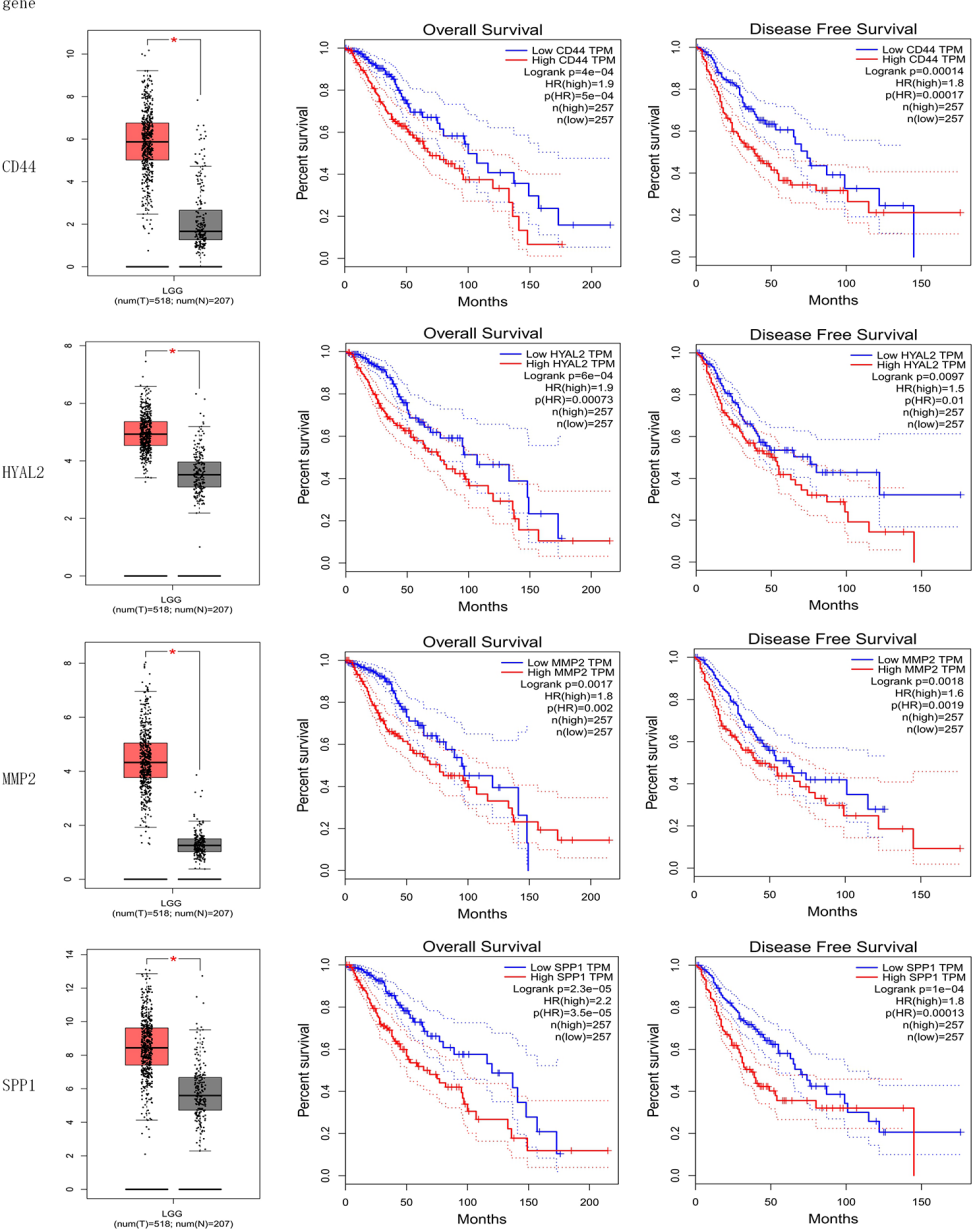
Identification of prognostic genes. CD44, HYAL2, MMP2, and SPP1 gene expression levels were significantly different in normal brain samples and LGG, and these four genes were associated with overall survival and disease-free survival of LGG. CD44, HYAL2, MMP2, and SPP1 were identified as prognostic genes consequently.

The result of the identification of prognostic genes was shown in **Figure 4** and **Supplementary Figure 1**. Five genes were differentially expressed in normal brain tissue and LGG, including CD44, HYAL2, MMP2, SPP1, RHOA; seven genes were significantly associated with OS of LGG, including CD44, HYAL2, MMP2, SPP1, ERBB2, MMP7, HMMR; eight genes were significantly associated with DFS of LGG, including CD44, HYAL2, MMP2, SPP1, ERBB2, MMP7, NANOG, HMMR. Therefore, CD44, HYAL2, MMP2, SPP1 were identified as prognostic genes (**Figure 4**), and seven genes were excluded from the gene signature (**Supplementary Figure 1**).

### Development and Validation of the Four-Gene Signature

Prognostic genes, including CD44, HYAL2, MMP2, SPP1, were used to construct a risk model:

risk score=(7.623E-03)∗CD44+(2.787E-02)∗HYAL2 + (8.357E-03)∗MMP2+(6.01E-04)∗SPP1

**Figure 5:**
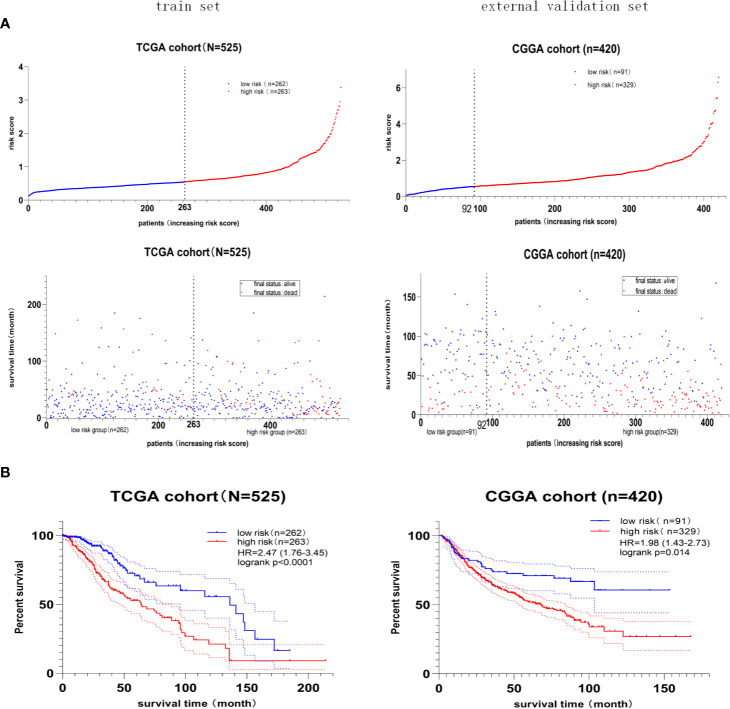
The prognostic value of the four-gene risk score model. **(A)** Risk score distribution and survival status of patients with LGG. LGG patients with a higher risk score possessed an unfavorable overall survival and higher death rate. **(B)** Kaplan–Meier survival curves of the high- and low-risk groups. The hazard ratio was 2.47 in the train set (p < 0.001), 1.98 in the external validation set (p = 0.014), demonstrating that the high-risk score predicted poor overall survival.

Furthermore, the median value of the risk score in the train set was 0.55. The patients were classified into the high- or low-risk group according to the train set’s median risk score. The distribution and status of OS (**Figure 5A**) showed that LGG patients with a higher risk score possessed an unfavorable overall survival and higher death rate. Besides, the Kaplan–Meier curve (**Figure 5B**) demonstrated that high-risk score predicted a worse prognosis, and the hazard ratio was 2.47 in the train set (p < 0.001), 1.98 in the external validation set (p = 0.014).

Lastly, the details of estimating the sensitivity and specificity of the risk model were shown in **Table 3**. For the 2-year survival prediction, the AUC values of the four-gene signature were 72.1 in the train set, 72.4 in the internal validation set, 67.5 in the external validation set. For 5-year survival prediction, the values of AUC in the training set, internal validation set, and external validation set were 63.3, 63.8, 67.1, respectively. For 8-year survival prediction, the values of AUC in the training set, internal validation set and external validation set were 65.5, 66.1, 68.5, respectively. For 10-year survival prediction, in the train set, internal validation set, and external validation set, the AUC values were 66.5, 68.1, 60.4. The AUC value in each set was over 60, and the accuracy of the risk model was validated in the validation set, indicating that the risk model had the potential to predict the prognosis of LGG.

**Table 3:** Accuracy of the risk model in the train set, internal validation set, and external validation set.

Year	Train set (TCGA)		Internal validation set (TCGA)		External validation set (CGGA)	
	AUC	95% CI	AUC	95% CI	AUC	95% CI
2	72.1	64.3–79.8	72.4	71.6–73.2	67.5	60.8-74.1
5	63.3	60.7–71.9	63.8	62.9–64.6	67.1	61.3-73.0
8	65.5	58.6–76.3	66.1	65.1–67.1	68.5	60.1-77.0
10	66.5	61.8–71.2	68.1	66.8–69.4	60.4	54.4-69.3

### Independent Prognostic Value of the Risk Score

As shown in **Table 4**, we performed the univariate analysis and multivariate analysis *via* SPSS software (version 20.0) to identify independent prognostic factors predicting OS in LGG patients. In the train set, risk score, age, radiotherapy, and IDH mutant were identified as independent prognostic factors. In the validation set, risk score, grade, IDH mutant, MGMT methylation were regarded as independent prognostic factors. The extent of resection of the tumor, the dose of radiation therapy, and the types of chemotherapy are essential factors in determining gliomas’ prognosis. To our surprise, radiotherapy showed the independent predictive value in the LGG cohort from TCGA; neither radiotherapy nor chemotherapy showed the independent predictive value in the LGG cohort from CGGA. Overall, the risk score and status of IDH had shown great independent prognostic value predicting OS in LGG patients; the high-risk score is an independent predictor of unfavorable OS (HR > 1), while IDH mutation status is an independent predictor of favorable OS in LGG (HR < 1). In one previous multivariate analysis conducted by Sanson et al., IDH mutation was also identified as an independent favorable predictor of glioma (49).

**Table 4:** Independent prognostic factors for OS of LGG.

LGG Cohort	Covariables	Univariate analysis			Multivariate analysis		
		HR	95%CI	p	HR	95%CI	p
Train set	Risk score (high vs low)	2.962	1.956–4.486	<0.001	2.108	1.325–3.353	0.002
	Age (≥60 *vs* <60)	3.01	1.794–5.052	<0.001	2.2	1.21–4	0.01
	Gender (male *vs* female)	1.045	0.706–1.546	0.825			
	Race (other *vs* white)	0.803	0.338–1.908	0.619			
	Grade (III *vs* II)	2.857	1.887–4.326	<0.001	1.565	0.956–2.561	0.075
	Radiotherapy (yes *vs* no)	3.528	2.118–5.877	<0.001	2.49	1.427–4.345	0.001
	IDH mutant (yes *vs* no)	0.153	0.054–0.434	<0.001	0.683	0.521–0.834	0.025
	Motor change (yes *vs* no)	0.882	0.539–1.444	0.618			
	Sensor change (yes *vs* no)	1.302	0.751–2.259	0.347			
	Seizure (yes *vs* no)	0.814	0.540–1.227	0.326			
	Headache (yes *vs* no)	0.897	0.586–1.373	0.617			
External validation set
	Risk score (high *vs* low)	2.377	1.449–3.898	<0.001	3.33	1.687–6.574	0.001
	Age (≥60 *vs* <60)	1.925	0.687–5.397	0.213			
	Gender (male *vs* female)	1.158	0.787–1.704	0.457			
	Grade (III vs II)	3.249	2.152–4.905	<0.001	3.666	2.161–6.218	<0.001
	Radiotherapy (yes *vs* no)	1.423	0.897–2.256	0.134			
	Chemotherapy (yes *vs* no)	1.188	0.781–1.808	0.42			
	IDH mutant (yes *vs* no)	0.441	0.272–0.713	<0.001	0.437	0.236–0.81	0.009
	MGMT methylation (yes *vs* no)	0.622	0.398–0.971	0.037	0.533	0.31–0.915	0.023

In the train set, four factors were identified as independent prognostic factors, including risk score, age, radiotherapy, IDH mutation. In the external validation set, four factors were identified as independent prognostic factors, including risk score, grade, IDH mutation, MGMT methylation. In conclusion, the risk score and status of IDH showed the independent prognostic value in two independent cohorts. P < 0.05 was considered statistically significant.

### The Relationship Between Gene Signature and LGG Subgroups

**Figure 6:**
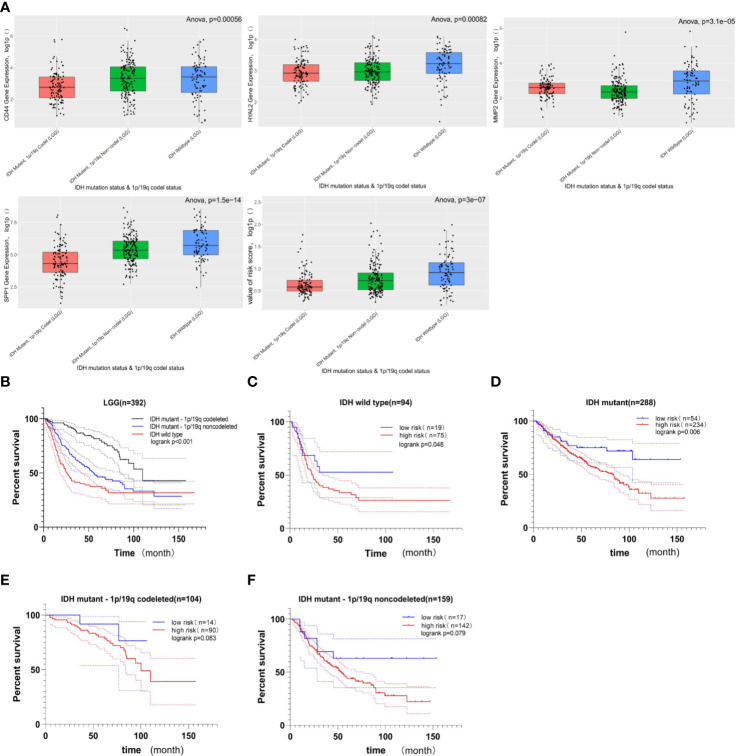
The relationship between gene signature and LGG subgroups based on IDH status and 1p/19q codeletion status. **(A)** Boxplots comparing the expression of CD44, HYAL2, MMP2, SPP1, and the value of risk score within LGG subtypes; the Y-axis represents the log1p-transformed gene expression or value of risk score. Among three subgroups of LGG, the LGG subtype with IDH wild-type status has the highest gene expression and risk score; in contrast, the LGG subtype with IDH mutant status and 1p/19q codeletion status has the lowest gene expression and risk score (all of p < 0.05, based on ANOVA test). **(B)** Kaplan–Meier survival curves of three LGG subgroups. The LGG subgroup with IDH wild-type status possesses the worst prognosis, and the subgroup with IDH mutation and 1p/19q codeletion has a better prognosis (logrank p < 0.001). **(C, D)** The prognostic value of gene signature in LGG when only the IDH status was considered. The Kaplan–Meier curve demonstrated that the gene signature can still divide LGG patients into high- and low-risk groups with distinct prognosis (logrank p < 0.05). **(E, F)** The prognostic value of gene signature in LGG subgroup when the IDH mutation and 1p/19q codeletion status were considered. In these two LGG subgroups, no significant difference in overall survival was observed between the high- and low-risk groups, but the higher risk score still suggested poor prognosis in a statistically significant trend toward (0.05< logrank p < 0.1).

The cohort from the CGGA database was divided into three subgroups based on IDH status and 1p/19q codeletion status; the expression of these four genes included in gene signature and the value of risk scores within three subgroups were shown in **Figure 6A**. Among these three LGG subgroups, the subgroup with IDH wild type had the highest gene expression level and risk score, and the subgroup with IDH mutant and 1p/19q codeletion had the lowest gene expression level and risk score among the three groups. In LGG patients, high-expression level of these four genes indicates poor prognosis (**Figure 4**), and high-risk score also indicates poor prognosis (**Figure 5**); it is reasonable to infer that subgroup with IDH wild type may have the worst prognosis, which further reflected distinct biological pattern between these three LGG subgroups.

Among these three LGG subgroups, we found that the LGG subgroup with IDH mutant and 1P /19q codeletion group had the best prognosis, while the LGG subgroup with IDH wild type had the worst prognosis (**Figure 6B**, log rank p < 0.05); this conclusion is consistent with previous studies (49–51). Then, we found that the gene signature still could divide LGG patients with the same IDH status into high- and low-risk groups with distinct prognosis (**Figures 6C, D**, log rank p < 0.05). What is more, when IDH mutant and 1p/19q codeletion were considered at the same time, no significant difference in OS was observed between high- and low-risk groups; there still exists a statistically significant trend toward that higher risk score suggesting the worst prognosis (**Figures 6E, F**, 0.05<logrank p < 0.1).

### High-Risk Scores Were Associated With Immune Checkpoints and Immune-Related Signaling Pathways

**Figure 7:**
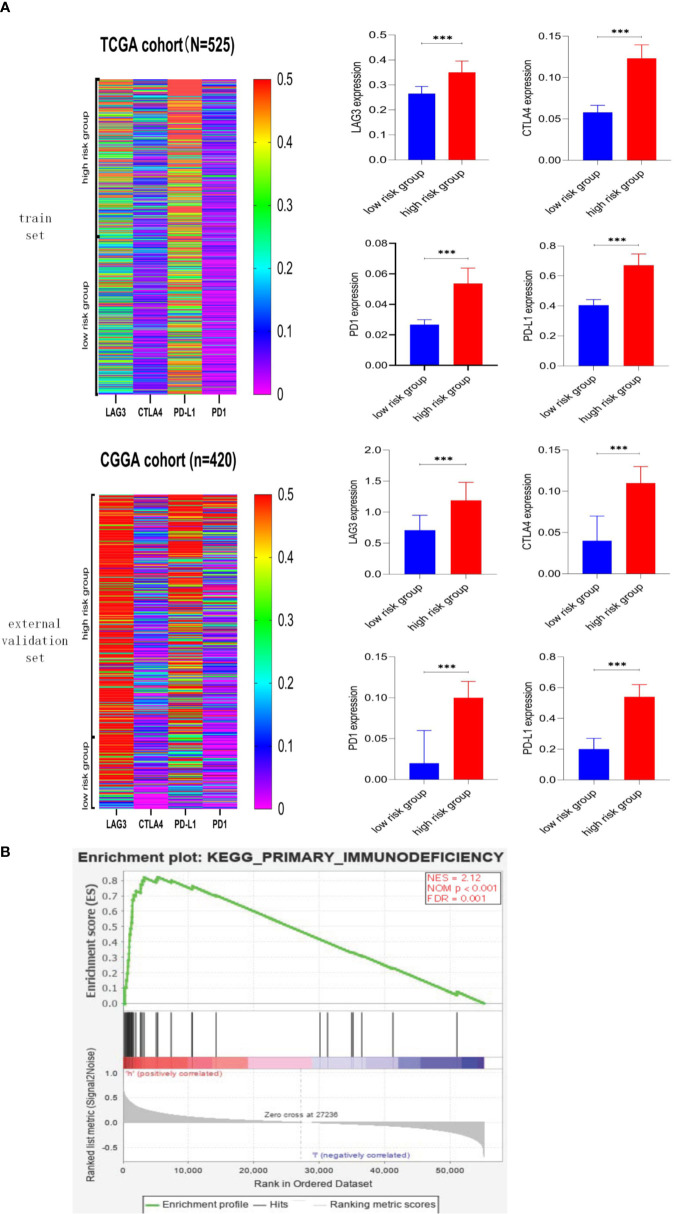
Association between risk score and immune checkpoint, immune-related pathway. **(A)** Heatmaps of immune checkpoints in LGG and the correlation between risk score and immune checkpoint. Immune checkpoints were mainly expressed in the high-risk score group, and the associations between higher risk score and higher expression level of immune checkpoints were significant (p < 0.001). **(B)** Gene Set Enrichment Analysis. Genes associated with high-risk phenotype were significantly enriched with the primary-immunodeficiency pathway. *** means p<0.001.

We explore the association between risk score and immune checkpoint *via* the heatmap and Mann–Whitney test, and detailed information was shown in **Figure 7A**. First, the heatmaps qualitatively demonstrated that the high-risk group possessed higher immune checkpoint expression, especially LAG3 and PD-L1. Then, the Mann–Whitney test verified that immune checkpoints were mainly expressed in the high-risk group rather than the low-risk group. The GSEA was performed to explore the potential immune-related pathways associated with the risk score. As shown in **Figure 7B**, genes associated with high-risk phenotype were significantly enriched with the primary-immunodeficiency pathway, and in the low-risk phenotype, we did not observe significantly enriched immune-related pathways. The association between risk score and immune checkpoints, the immune-related pathway may be able to explain why the LGG patients with higher risk scores possess shorter survival time and higher death rates (**Figure 5A**).

### Tools for Predicting Survival Probability

**Figure 8:**
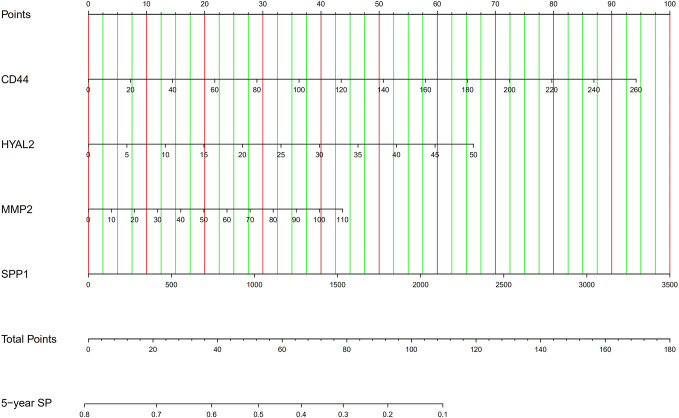
The nomogram for predicting 5-year survival probability of LGG. SP, survival probability.

Firstly, we developed an excel table (**Supplementary Table 1**); it was just needed to submit the value of gene expression to predict survival probability, and the excel table was capable of calculating risk score. Risk score >0.55 indicates a poor prognosis. Then, the nomogram for predicting 5-year survival probability was shown in **Figure 8**. These tools may accurately predict the survival probability of LGG patients.

## Discussion

The survival time for patients with LGG varies widely, ranging from 1 year to 15 years (52); complete resection of LGG still is a challenge because of its invasive nature, and LGG is prone to progress glioblastoma (53). Therefore, obtaining an accurate prognosis at the early stage of the tumor can help improve the clinical outcome of patients; it remains an issue to elucidate the underlying mechanisms behind LGG progression and to identify molecular pathways for special treatment.

The prognostic value of CD44 expression in gliomas was inconsistent. Three studies suggested that high expression of the CD44 gene was significantly associated with poor prognosis in glioma patients (28–30), and two studies suggested that CD44 gene expression was not significantly associated with prognosis (31, 32). In contrast, one study suggested that higher CD44 gene expression was associated with a better prognosis in GBM (33). In the present study, we confirmed that the CD44 gene was the DEG of LGG and was highly expressed in LGG (**Figure 2** and **Figure 4**). The GO analysis of the CD44 gene and its related genes showed that these genes play a role in many biological processes, including extracellular matrix disassembly, cell adhesion, plasma membrane, extracellular exosome, protein binding, hyaluronic acid-binding (**Figures 3B, D**). Previous studies have shown that the expression of hyaluronic acid receptor CD44 and its adherence to hyaluronic acid are involved in aggressiveness (54). The expression level of CD44 is related to the histopathological grade of gliomas, and the monoclonal anti-CD44 antibody is capable of inhibiting the migration of glioma cells (28). CD90, another tumor stem cell marker in gliomas, plays an essential role in tumor migration, dasatinib response, and temozolomide-resistance (55, 56). The expression of CD90 is increased in a grade-dependent manner, which is similar to CD44; CD90 is capable of distinguishing grade III/IV gliomas from grade I/II gliomas and normal brain tissue because CD90 mainly expresses in high-grade gliomas and rarely expresses in low-grade gliomas and normal brain tissue (57). The alternation of the tumor immunological environment also influences the expression of CD44. In the GL261 murine glioma model, low expression of CD44 and CD122 was found in CD4 + and CD8 + T cells, but an increased proportion of CD44 + T cells was found in double-negative (CD4 - and CD8 -) T cells (58). The KEGG pathway analysis (**Figure 3E**) showed that CD44-related genes regulate the occurrence and development of tumors through multiple tumor-related pathways, which may become the target pathways for the treatment of gliomas and provide ideas for the precise treatment strategies of patients.

Among the CD44-related genes, CD44, HYAL2, MMP2, and SPP1 were identified as prognostic genes of LGG since these four genes were DEG related to OS and DFS. These four genes were used to develop a CD44-related gene signature for the prediction prognosis of LGG patients. In one previous research, Yan et al. (59) have established a CD133-related gene signature for predicting glioblastoma prognosis; the CD133-related signature successfully distinguishes GBM from LGG, and the CD133-related gene signature defines a new subtype of GBM with shorter survival time. In colorectal cancer, high expression of HYAL1 and HYAL2 can inhibit tumor metastasis (60), but in triple-negative breast cancer, high expression of HYAL2 genes is associated with shorter disease-free survival, higher tumor recurrence rate, and higher tumor metastasis rate (61). MMP2 is involved in the invasion of thyroid tumor cells, and its expression is regulated by the ERK and JNK pathways (62, 63). In colorectal cancer, upregulated SPP1 is associated with poor survival outcomes (64); miR-340 can inhabit the phosphatidylinositol 3-kinase/protein kinase B pathway, and miR-340 also contribute to the suppression of proliferation, migration, and invasion of gastric cancer *via* reducing the expression of SPP1 (65).

In the present study, we constructed a novel four-gene signature (including CD44, HYAL2, MMP2, SPP1) for predicting the prognosis of LGG patients; the predictive gene model was externally validated by CGGA-LGG set. The gene signature’s prognostic accuracy was estimated by AUC value, which was higher than 0.6 in the development set, internal validation set, and external validation set (**Table 3**). AUC > 0.5 indicates a predictive role in patients with LGG. The gene signature could effectively classify patients into low-risk and high-risk groups with distinct outcomes (**Figure 5**). Additionally, the independent prognostic value of the four-gene signature was verified in TCGA-LGG set (HR = 2.108, p = 0.002) and in CGGA-LGG set (HR = 3.33, p = 0.001), indicating higher risk score was an adverse prognostic factor for patients with LGG. The present study also identified IDH mutation as an independent predictor of favorable OS, and this finding is consistent with previous research (49). Although in the multivariate analysis, radiotherapy’s independent predictive value was not consistent, and chemotherapy did not show an independent predictive value. In the survival of LGG, no survival difference was observed between lower dosage and higher dosage of radiotherapy; a lower dosage of radiotherapy exhibits fewer side effects (66, 67). Studies have shown that the combination of radiotherapy and chemotherapy can improve outcomes for LGG (66, 67), but there is no significant survival difference between radiotherapy alone and chemotherapy alone (68, 69).

The updated classification of tumors of CNS divides the LGG into three subgroups based on IDH mutation status and 1p/19q codeletion status, and these three types of LGG vary in genetic characteristics and prognosis (2). The function of the IDH enzyme is to catalyze the transformation from isocitrate into *α*-keto-*β*-carboxy glutamic acid, and the mutant IDH consumes *α*-keto-*β*-carboxy glutamic acid for D-2-hydroxyglutarate synthesis in an NADPH-dependent manner (70). Numerous studies have shown that mutations in IDH affect many biological processes (71–76), including cellular metabolism, epigenetic shift, genomic instability, and redox Q[CE] homeostasis. IDH mutation has become the essential molecular in the diagnosis of gliomas. In the present study, we divided the LGG cohort from the CGGA database into three groups, including IDH-wild type LGG, IDH-mutant and 1p/19q non-codeletion LGG, and IDH-mutant and 1p/19q codeletion LGG, and then we explored the relationship between gene signature and these three subgroups. We found that gene signatures were mainly presented in IDH-wild type LGG patients who also possess a higher risk score. The Kaplan–Meier curve suggested an unfavorable prognosis in IDH-wild type LGG patients; IDH-mutant gliomas have a favorable prognosis, especially the IDH-mutant glioma combined with 1p/19q codeletion; this conclusion is consistent with previous research. What is more, the gene signature was capable of dividing LGG patients with the same IDH status into high- and low-risk groups (**Figures 6C, D**, p < 0.05). However, when 1p /19q codeletion status was also taken into account, the ability to distinguish high- and low-risk populations was weakened, and there was no statistical significance, only a trend (**Figures 6E, F**, p < 0.1).

Targeting the tumor immune checkpoints may be a novel strategy to kill tumor cells. Therefore, the association between risk scores and the expression of immune checkpoints was the focus of our research. We described the expression profiles of four immune checkpoints in LGG, PD-L1, and LAG3 were apparently expressed in patients with a higher risk score, and higher risk score was intimately associated with higher expression of immune checkpoints. There is growing evidence that IDH mutation could suppress tumor-infiltrating lymphocytes’ activation and create an immunosuppressive tumor microenvironment (77, 78). Compared with IDH wild type gliomas, IDH mutant gliomas showed lower expression levels of PD-1 and PD-L1 (79–81). The lower expression of checkpoints in IDH-mutant gliomas is due to D-2-hydroxyglutarate, an essential product of IDH mutant cancer, which results in epigenetic regulation *via* DNA methylation (79, 80). The combination of PD-L1 expressed in tumor cells and PD-1 expressed in immune cells can inhibit T cells’ activation, inhibit the monitoring function of immune cells, and contribute to the immune escape of tumor cells (82). Current studies have shown that PD-L1 is not only a prognostic biomarker for glioma but also a promising therapeutic target for glioma (83). CTLA-4 and LAG3 are another two immune checkpoints and play an essential role in activating T cells (84, 85).

What is more, the expression of immune checkpoints correlates with immunotherapy’s immune response (86–88). The patients with high-risk scores had higher immune checkpoint expression, suggesting that these patients may be more sensitive to immunotherapy. However, the factors that determine the sensitivity of immunotherapy include the expression level of immune checkpoints and the type and number of tumor-infiltrating lymphocytes. Compared with glioblastoma, the number of CD8^+^ T cells in LGG was significantly reduced, and the reduction of CD8 cells suggested a tolerance to immunotherapy (89). The genetic and genomic alternations in LGG may influence immunotherapy sensitivity *via* recruiting T cells and microglia (90). The GSEA analysis showed that genes associated with high-risk phenotype were significantly enriched with primary immunodeficiency pathway (**Figure 7B**); the previous studies have shown that patients with primary immunodeficiency tend to have a higher incidence of cancer because of genomic instability due to defective DNA repair mechanisms (91, 92).

In adopting the conclusions of this study, several limitations also need to be considered. First of all, the relationship between the four genes included in the gene prediction model and the biological mechanism of LGG has not been studied clearly. Second, we found a positive correlation between the risk score and the expression of immune checkpoints, and a higher risk score is associated with the activation of immunosuppression related pathways, resulting in the patients with higher risk score having a higher mortality rate and shorter overall survival. However, more follow-up studies are needed to verify the relationship between the risk scores and the immune checkpoints to identify the specific mechanism of genes-regulation of the immune-related signaling pathway.

## Conclusion

CD44, a tumor stem cell biomarker, is upregulated in LGG, and four CD44-related genes with the prognostic value may become prognostic markers and therapeutic targets for low-grade gliomas. These four genes are used to construct a gene signature, which can effectively divide LGG patients into high- and low-risk groups with the distinct outcome; risk score, and status of IDH were independent predictors in LGG. The present analysis further confirmed distinct biological patterns between oligodendroglioma, IDH-mutant astrocytoma, and IDH-wild type astrocytoma. The gene signature can divide the LGG patients with the same IDH status into high- and low-risk groups. This study also found that higher mortality and shorter survival in the high-risk group may be associated with high expression of immune checkpoints of tumor cells and may be associated with immunosuppressive pathways.

## Data Availability Statement

The datasets presented in this study can be found in online repositories. The names of the repository/repositories and accession number(s) can be found in the article/**Supplementary Material**.

## Author Contributions

YX, JH, GC, and XR: conception of the study, formal analysis, manuscript preparation, and writing. HD, CH, GC, and HW: academic instruction, funding acquisition, manuscript reviewing, and editing. XY and YZ: resources, software. XZ and ZW: data extraction, data curation, constructive discussions. All authors contributed to the article and approved the submitted version.

## Funding

This work was supported by the National Natural Science Foundation Youth Fund (grant number 30600637), the China Postdoctoral Science Foundation (grant numbers 2014M561207 and 2019T120195), and the Shanxi Scholarship Council of China (grant number 2011-096,2016-key project 4).

## Conflict of Interest

The authors declare that the research was conducted in the absence of any commercial or financial relationships that could be construed as a potential conflict of interest.

## Supplementary Material

Supplementary Figure 1 Identification of prognostic genes. Seven genes were not identified as prognostic gene, including ERBB2, HMMR, MMP7, MMP9, NANOG, RHOA, SELE.

Supplementary Table 1 Excel table for predicting 2-, 5-, 8- and 10-year survival probability of LGG patient.

## Abbreviations

LGG, lower grade glioma; GEO, Gene Expression Omnibus; PPI, protein–protein interaction; OS, overall survival; DFS, disease-free survival; CSC, cancer stem cell; DEG, differentially expressed gene; GO, gene ontology; KEGG, Kyoto Encyclopedia of Genes and Genomes; CC, cellular components; BP, biological processes; MF, molecular functions; TCGA, The Cancer Genome Atlas; CGGA, Chinese Glioma Genome Atlas; AUC, area under the receiver operating characteristic curve; PD1, programmed cell death protein 1; PD-L1, Programmed cell death 1 ligand 1; CTAL4, Cytotoxic T Lymphocyte Antigen 4; LAG3, Lymphocyte-activation gene 3; GSEA, Gene Set Enrichment Analysis.
