# Supplementary figures and images for: A Novel Four-Gene Signature Associated With Immune Checkpoint for Predicting Prognosis in Lower-Grade Glioma
Source: Front Oncol. 2020 Oct 30;10:605737. doi: 10.3389/fonc.2020.605737 (PMC7769121; doi:10.3389/fonc.2020.605737)

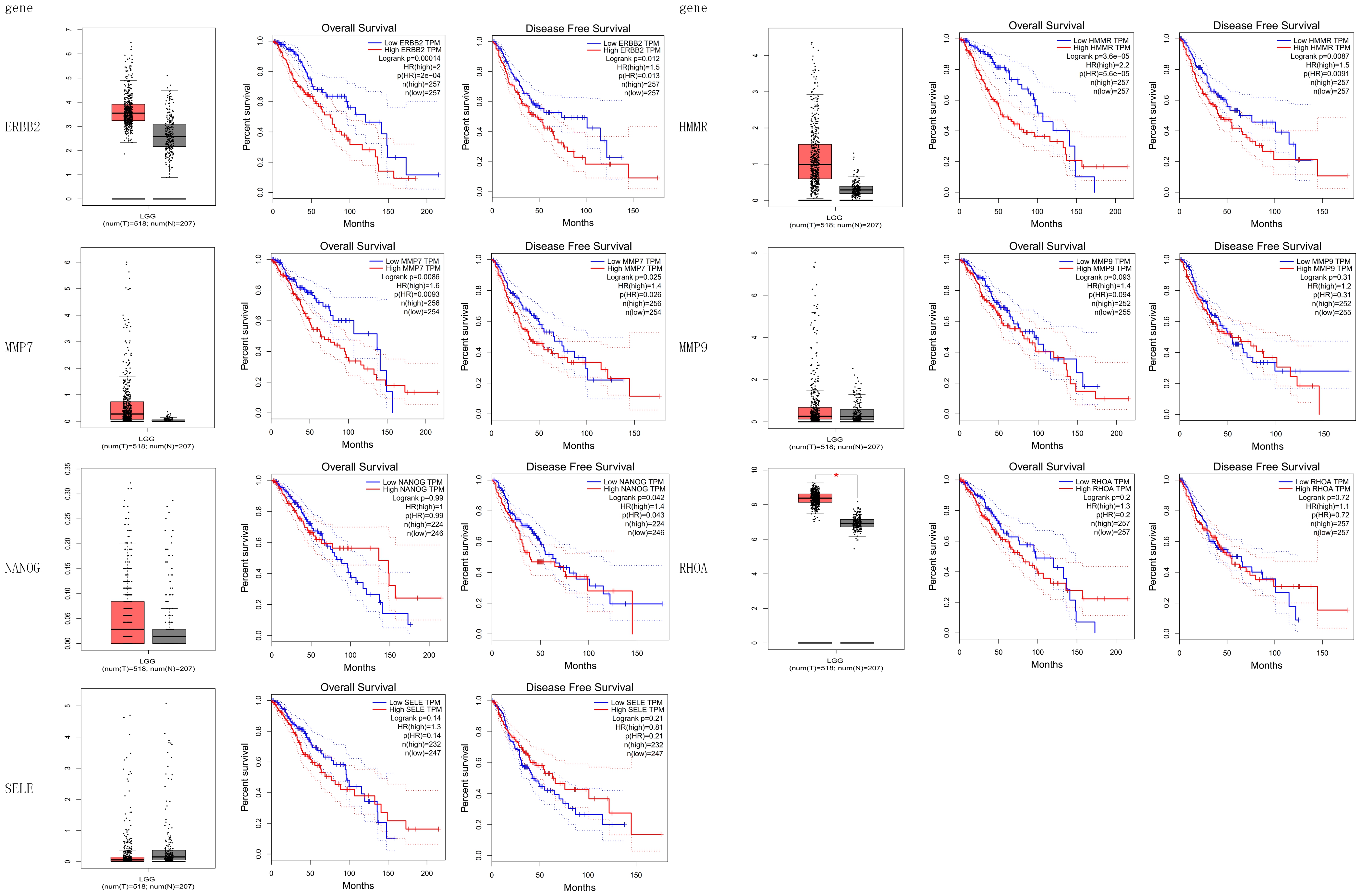

Supplement: Supplementary Figure 1 — Identification of prognostic genes. Seven genes were not identified as prognostic gene, including ERBB2, HMMR, MMP7, MMP9, NANOG, RHOA, SELE. [file Image_1.jpeg]
